# Local Administration of Minocycline Improves Nerve Regeneration in Two Rat Nerve Injury Models

**DOI:** 10.3390/ijms241512085

**Published:** 2023-07-28

**Authors:** Owein Guillemot-Legris, Gedion Girmahun, Rebecca J. Shipley, James B. Phillips

**Affiliations:** 1 UCL Centre for Nerve Engineering, London WC1N 1AX, UK; gedion.girmahun.21@ucl.ac.uk (G.G.); rebecca.shipley@ucl.ac.uk (R.J.S.); jb.phillips@ucl.ac.uk (J.B.P.); 2 UCL School of Pharmacy, London WC1N 1AX, UK; 3 UCL Mechanical Engineering, London WC1E 7JE, UK

**Keywords:** nervous system, nerve regeneration, electrophysiology, CMAP, repair phenotype, Schwann cells, fibrin, nerve repair

## Abstract

Peripheral nerve injuries are quite common and often require a surgical intervention. However, even after surgery, patients do not often regain satisfactory sensory and motor functions. This, in turn, results in a heavy socioeconomic burden. To some extent, neurons can regenerate from the proximal nerve stump and try to reconnect to the distal stump. However, this regenerating capacity is limited, and depending on the type and size of peripheral nerve injury, this process may not lead to a positive outcome. To date, no pharmacological approach has been used to improve nerve regeneration following repair surgery. We elected to investigate the effects of local delivery of minocycline on nerve regeneration. This molecule has been studied in the central nervous system and was shown to improve the outcome in many disease models. In this study, we first tested the effects of minocycline on SCL 4.1/F7 Schwann cells in vitro and on sciatic nerve explants. We specifically focused on the Schwann cell repair phenotype, as these cells play a central role in orchestrating nerve regeneration. Finally, we delivered minocycline locally in two different rat models of nerve injury, a sciatic nerve transection and a sciatic nerve autograft, demonstrating the capacity of local minocycline treatment to improve nerve regeneration.

## 1. Introduction

Injuries to peripheral nerves are common, and following surgical intervention, only 50% of treated individuals regain satisfactory function [1]. This can result in life-long pain and disabilities, and because nerve injuries disproportionally affect a younger population of working age patients, the associated healthcare, rehabilitation, social, and unemployment costs are very high [2,3]. Depending on the scale of nerve injury, the surgical option can involve directly suturing the disconnected stumps (for small gaps) with the use of fibrin glue to strengthen the repair [4,5]. Regarding longer gaps, where direct suturing would cause excessive tension, the gold standard procedure to repair the injury is an autograft, where nerve tissue is harvested from elsewhere in the patient and used as a bridge to connect the stumps of the damaged nerve [2,3]. Here again, fibrin glue is very often used to strengthen the sutures [4,5]. To some extent, neurons are able to regenerate from the proximal stump of a repaired nerve into the distal stump. However, this regeneration is limited, takes time, and can be unsuccessful, specifically in the context of long gap repairs [2,3]. Therefore, there is a clear unmet clinical need for the acceleration and improvement of nerve regeneration.

In the context of nerve regeneration, no drug therapies are used routinely to promote nerve regeneration following repair surgery. Minocycline is a tetracyclic antibiotic with bacteriostatic activity, which has been widely studied for its immunomodulatory and anti-inflammatory effects. It has been shown that minocycline was able to prevent microglia proliferation (rat primary cells) upon N-methyl-D-aspartate challenge and reduced both interleukin-1β and nitric oxide release [6]. Interleukin-1β release was also reduced in lipopolysaccharide-activated THP-1 cells (monocytic cell line) upon minocycline treatment [7]. In the context of the nervous system, minocycline was able to improve outcomes associated with central nervous system disorders in several animal models. For instance, minocycline decreased the number of infarcts in models of ischaemic stroke; decreased axonal, neuronal, and oligodendrocyte loss in models of spinal cord injury; and was able to decrease lesion size and improve functional outcomes in models of traumatic brain injury [8,9]. The effects of minocycline were explored mostly in the central nervous system, whereas in the peripheral nervous system, there have been relatively few studies, and in those, it was administered systemically and led to mixed effects in terms of regeneration [10,11].

Peripheral nerve regeneration is a complex process requiring precise and timely crosstalk between several cell types. Schwann cells represent a key player during the whole duration of the process. Following injury, these cells undergo profound changes, adopting a repair phenotype able to guide and support axon growth and encourage regeneration [12]. Macrophages are another important cell type in nerve regeneration as they initiate the repair process in response to hypoxia and orchestrate the migration of endothelial cells and Schwann cells [13,14].

In this study, we first evaluated the effects of minocycline on Schwann cells and, more specifically, on markers associated with the repair phenotype. Using RT-qPCR and immunofluorescence, we found that several key markers of the repair phenotype were increased following minocycline treatment in vitro. We then used two in vivo rat models of nerve regeneration to study the impact of minocycline: sciatic nerve transection with direct repair and sciatic nerve autograft. These represent the two main scenarios encountered in the clinic to repair nerve transections, and in each case, the minocycline was delivered locally using fibrin gel. This route of administration represents a viable option [15,16] as it does not add time to the surgery, will deliver the drug locally (avoiding potential systemic side effects), delivers a specific quantity of a drug, and targets the specific region where the drug is needed. We assessed markers associated with nerve regeneration in the sciatic and common peroneal nerves of these animals and conducted an assessment of the motor function of the gastrocnemius and tibialis anterior muscles using electrophysiology.

## 2. Results

### 2.1. Minocycline Decreases RAW 264.7 Cell Activation

Minocycline effects have been evaluated in vitro on several different cell types using a wide range of concentrations [17,18,19]. Myeloid-derived cells, such as microglia, monocytes, and macrophages, have been one of the most studied in this context. In order to select specific doses of minocycline for further in vitro experiments, we evaluated the effects of three commonly used concentrations shown to decrease the expression of factors in myeloid cell activation [7,20,21,22]. RAW 264.7 cells (mouse macrophage-like cell line) were treated for 24 h with minocycline at 1, 20, or 40 µM or with the vehicle. The mRNA expression of Ccl2 and Cxcl2 was significantly decreased when treated with minocycline at 20 and 40 µM, while Ptgs2 and Ccl3 expression was significantly decreased at the three concentrations used when compared with vehicle-treated cells (Figure S1). We also found that Tnf expression was not altered. Finally, the levels of CCL2 in the media were significantly decreased with minocycline treatment at 20 and 40 µM, mirroring the variations observed for Ccl2 mRNA expression (Figure S1). Given these results, we elected to use 1 and 40 µM for further in vitro testing.

### 2.2. Minocycline Alters the Expression of Key Factors Associated with Schwann Cell Repair Phenotype in SCL 4.1/F7 Cells

**Figure 1:**
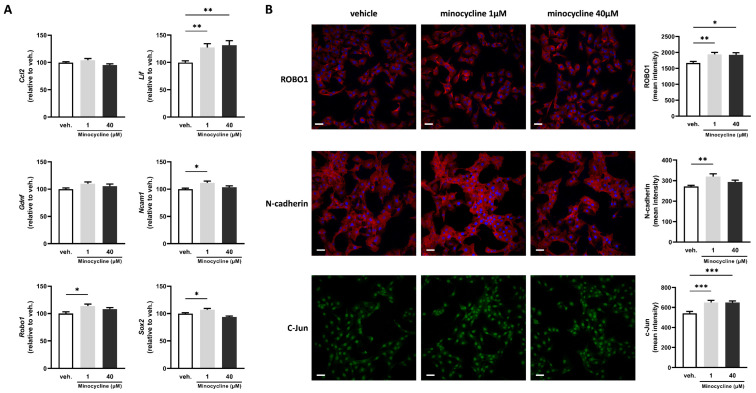
Minocycline induces the expression of key factors associated with Schwann cell repair phenotype in SCL 4.1/F7 cells. (**A**) mRNA expression of Ccl2, Lif, Gdnf, Ncam1, Sox2, and Robo1 in SCL 4.1/F7 Schwann cells treated with minocycline (1, 40 µM) or vehicle (veh.) for 24 h assessed by RT-qPCR. The expression of the control group (veh.) was set at 100. N = 3 in quadruplicate. (**B**) Mean intensity of ROBO1, N-cadherin, and c-Jun quantified by immunofluorescence in SCL 4.1/F7 Schwann cells treated with minocycline (1, 40 µM) or vehicle (veh.) for 24 h. N = 3 with five technical replicates. Scale bar = 50 µm. Data are mean ± sem. One-way ANOVA with Dunnett’s post hoc test or Kruskal–Wallis with Dunn’s post hoc test depending on the normality of the distribution. * *p* <  0.05, ** *p* <  0.01, and *** *p* < 0.001.

Schwann cells represent a key cell type in the context of nerve regeneration. Indeed, they orchestrate complex and time-specific processes that are central to successful regeneration. One unique specific feature of Schwann cells is their ability to switch toward a “repair” phenotype that can promote axonal regeneration, neuron survival, and target reinnervation [23]. Therefore, we wanted to assess the effects of minocycline on factors associated with Schwann cell repair phenotype. SCL 4.1/F7 cells (rat Schwann cell line) were treated for 24 h with minocycline at 1 or 40 µM or with the vehicle. The expression of Ncam1, Sox2, and Robo1 was significantly increased at 1 µM compared with vehicle-treated cells (by 10%, 7%, and 14%, respectively) (Figure 1A).

Interestingly, the expression of Lif, a cytokine found to be beneficial in the context of nerve regeneration [24], was increased at the two minocycline concentrations used (27% increase at 1 μM and 32% increase at 40 μM). Finally, we found no change in the expression of Ccl2 and Gdnf (Figure 1A). To further characterise minocycline-induced changes in Schwann cell repair phenotype markers, we also used immunofluorescence to detect changes in protein levels. We found that the mean intensity of ROBO1 was significantly increased at both 1 and 40 µM compared with vehicle-treated cells (16% and 15%, respectively) (Figure 1B). N-cadherin was significantly increased at 1 µM (by 17%), while c-Jun mean intensity was increased at both concentrations (20% increase for both) (Figure 1B).

### 2.3. Minocycline Alters the Expression of Key Factors Associated with Schwann Cell Repair Phenotype in Sciatic Nerve Explants

**Figure 2:**
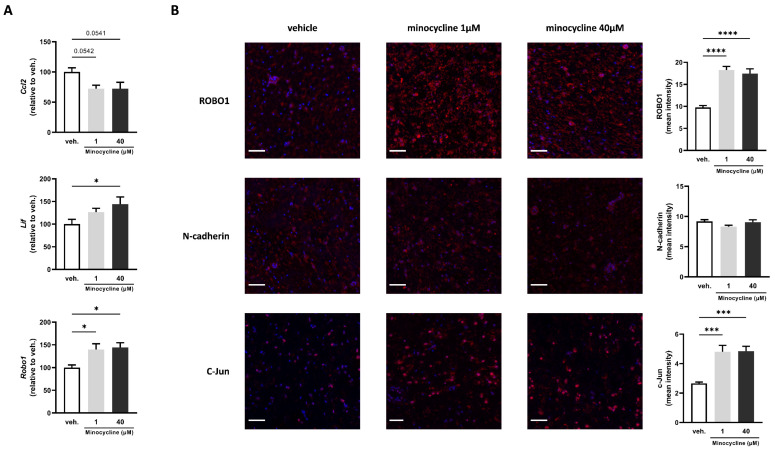
Minocycline induces the expression of key factors associated with Schwann cell repair phenotype in sciatic nerve explants. (**A**) mRNA expression of Ccl2, Lif, and Robo1 in sciatic nerve explants treated with minocycline (1, 40 µM) or vehicle (veh.) for 24 h assessed by RT-qPCR. The expression of the control group (veh.) was set at 100. N = 5. (**B**) Mean intensity of ROBO1, N-cadherin, and c-Jun quantified by immunofluorescence in sciatic nerve explants treated with minocycline (1, 40 µM) or vehicle (veh.) for 24 h. N = 6–8. Scale bar = 50 µm. Data are mean ± sem. One-way ANOVA with Dunnett’s post hoc test or Kruskal–Wallis with Dunn’s post hoc test depending on the normality of the distribution. * *p* <  0.05, *** *p* <  0.001, and **** *p* < 0.0001 or indicated *p*-value.

In order to explore further the effects of minocycline on the peripheral nervous system, we elected to use rat sciatic nerve explants. The use of primary tissue provides an effective way to study key aspects of pathophysiological processes encountered in vivo since the three-dimensional organisation of the tissue and cell–cell interactions is maintained. This ex vivo setup is characterised by the degeneration of axons within the tissue due to the separation of the nerve from the neuronal cell bodies located in the dorsal root ganglia or spinal cord, thus replicating one of the central aspects encountered in nerve injury. Rat sciatic nerve explants were treated for 24 h with minocycline at 1 or 40 µM or with the vehicle. The expression of Ccl2 displayed a trend towards a decrease at 1 µM and 40 µM compared with vehicle-treated sciatic nerve explants (by 28% for both) (Figure 2A).

The expression of Lif was only significantly increased at 40 µM compared with vehicle-treated sciatic nerve explants (by 44%). Finally, Robo1 expression was significantly increased at 1 µM and 40 µM compared with vehicle-treated sciatic nerve explants (by 40% and 44%, respectively) (Figure 2A). To further assess the effects of minocycline on key markers of Schwann cell repair phenotype, we used immunofluorescence to detect changes in protein levels. We found that the mean intensity of ROBO1 was significantly increased at both 1 and 40 µM compared with vehicle-treated sciatic nerve explants (87% and 79%, respectively) (Figure 2B). We found no changes in N-cadherin mean intensity. Finally, c-Jun mean intensity was increased significantly at both concentrations (81% and 82%, respectively) (Figure 2B).

### 2.4. Minocycline Locally Delivered in Fibrin Gel Improves Nerve Regeneration following Sciatic Nerve Transection and Direct Repair

**Figure 3:**
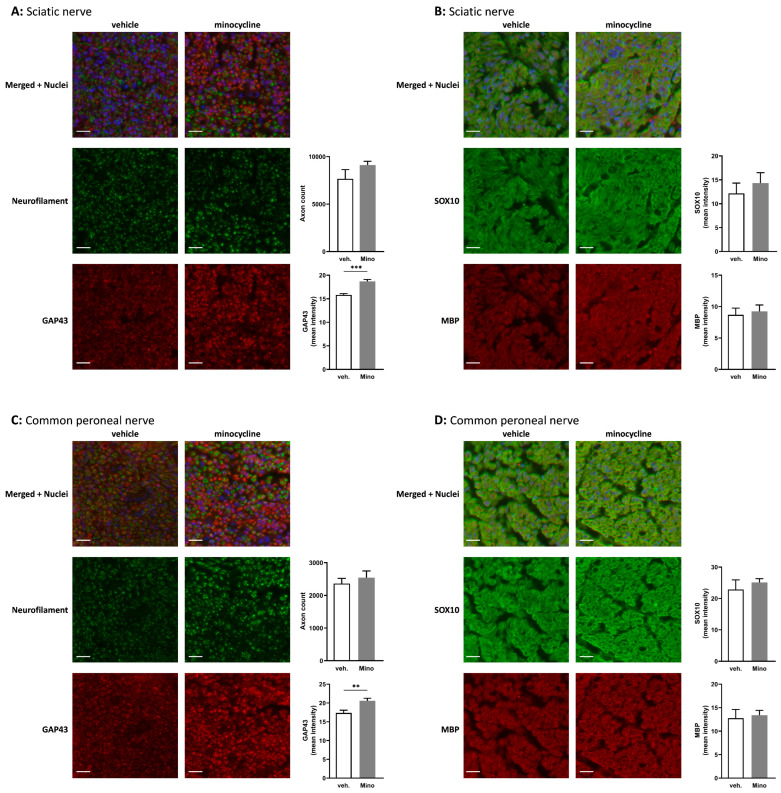
Effect of locally delivered minocycline on histological features of nerves following transection and direct repair. The axon count was performed using immunofluorescence detection of neurofilament medium and heavy chains in rats that underwent nerve transection and repair, treated 7 days later with fibrin gel and water (vehicle) or fibrin gel and minocycline (Mino) in (**A**,**B**) the sciatic nerve, and (**C**,**D**) the common peroneal nerve 35 days after surgery (or 28 days after treatment). The mean immunofluorescence intensities of GAP43 (**A**,**C**), SOX10, and MBP (**B**,**D**) were quantified in (**A**,**B**) the sciatic nerve and (**C**,**D**) the common peroneal nerve. Scale bar = 20 µm. Data are mean ± sem. N = 5–7 per group. Two-tailed unpaired *t*-test or a Mann–Whitney test were used depending on the normality of the distribution. ** *p* <  0.01 and *** *p* < 0.001.

Minocycline has been studied for its immunomodulatory and anti-inflammatory effects, and we showed that it decreased RAW 264.7 cell activation (Figure S1). It is also able to increase key markers associated with Schwann cell repair phenotype (Figure 1A,B). Therefore, it seemed that minocycline could be beneficial in the context of nerve regeneration. Seven days after sciatic nerve transection and direct repair, rats were treated with vehicle (water) or 100 µg of minocycline delivered at the repair site in 20 µL of fibrin gel (Figure S2A). This dose was selected based on animal models of central nervous system pathologies where minocycline was administered locally [25,26,27]. Thirty-five days after the nerve transection, sciatic and common peroneal nerves were harvested. Immunofluorescence assessment showed no significant difference in axon counts between vehicle-treated or minocycline-treated animals (Figure 3A,C).

However, growth-associated protein-43 (GAP43), a protein expressed at high levels during regeneration of the peripheral nervous system [28], was significantly increased in both sciatic and common peroneal nerves in animals locally treated with minocycline (Figure 3A,C). Finally, SOX10, an essential Schwann cell transcription factor involved in PNS myelination [29,30], displayed no change in the sciatic nerve or the common peroneal nerve (Figure 3B,D).

**Figure 4:**
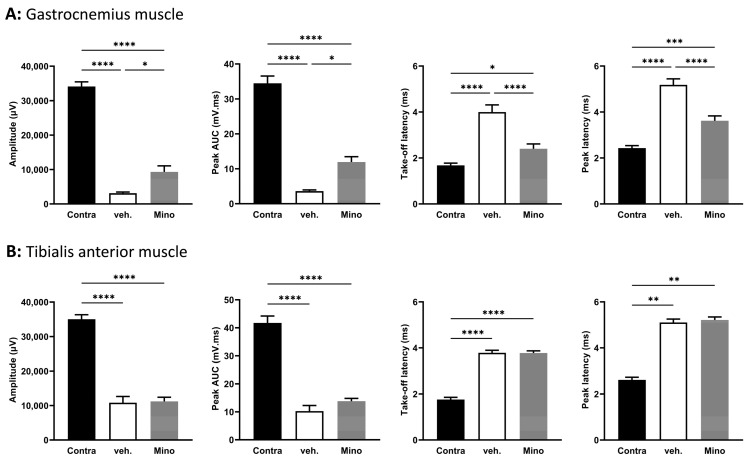
Minocycline locally delivered in fibrin gel improves compound muscle action potential parameters after sciatic nerve transection. Thirty-five days after surgery (so, twenty-eight days after treatment), electrophysiology was performed and compound muscle action potentials were recorded in (**A**) the gastrocnemius muscle and (**B**) the tibialis anterior muscle. Four parameters were quantified from the CMAP traces: amplitude, peak AUC, take-off latency, and peak latency. Data are mean ± sem. N = 5–7 per group. One-way ANOVA with Šídák’s post hoc test was used. * *p* <  0.05, ** *p* <  0.01, *** *p* < 0.001, and **** *p* < 0.0001.

To assess regeneration, we studied functional recovery thirty-five days post transection (so, twenty-eight days after local treatment) using electrophysiology. We recorded compound muscle action potential (CMAP) in the gastrocnemius muscle (Figure 4A) and the tibialis anterior muscle (Figure 4B).

In the gastrocnemius muscle, both amplitude and peak area under the curve (AUC) were significantly higher in minocycline-treated animals compared with vehicle-treated animals (2.9-fold and 3.3-fold, respectively) (Figure 4A). For latencies, minocycline-treated animals displayed lower values than vehicle-treated animals (1.67-fold decrease in take-off latency and 1.43-fold decrease in peak latency) (Figure 4A). In the tibialis anterior muscle, the CMAP traces did not show any improvement in minocycline-treated animals compared to the vehicle (Figure 4B). These results indicate an improvement in motor function at thirty-five days in the gastrocnemius muscle but not in the tibialis anterior muscle for animals treated with minocycline.

### 2.5. Minocycline Locally Delivered in Fibrin Gel Improves Nerve Regeneration in Sciatic Nerve Autograft

**Figure 5:**
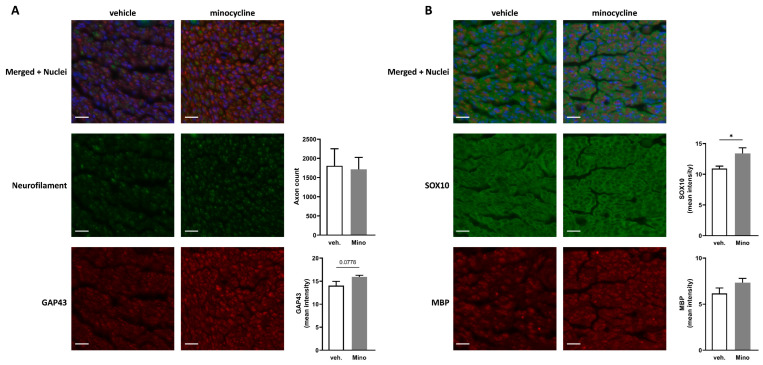
Effect of locally delivered minocycline on histological features of the common peroneal nerve following sciatic nerve autograft. The axon count was performed using immunofluorescence detection of neurofilament medium and heavy chains in rats that underwent nerve autograft repair treated with fibrin gel and water (vehicle) or fibrin gel and minocycline (Mino) in the common peroneal nerve 52 days after surgery (**A**). The mean immunofluorescence intensities of GAP43 (**A**), SOX10, and MBP (**B**) were quantified in the common peroneal nerve. Scale bar = 20 µm. Data are mean ± sem. N = 5–6 per group. Two-tailed unpaired t-test or a Mann–Whitney test were used depending on the normality of the distribution. * *p* <  0.05 or indicated value.

Using a different model in which a 1 cm sciatic nerve autograft was used to repair a transection, minocycline (or vehicle) was applied at the two suture sites in 10 µL of fibrin gel per site (SI2B). The total dose of minocycline locally delivered was 100 µg (50 µg per suture site). An assessment of neurofilament-positive neurites in the common peroneal nerve after 52 days showed no difference in axon counts between vehicle-treated or minocycline-treated animals (Figure 5A).

**Figure 6:**
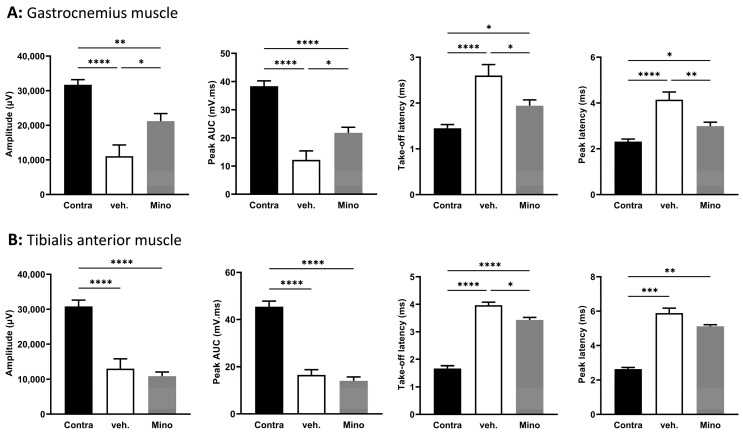
Minocycline locally delivered in fibrin gel improves compound muscle action potential parameters after a sciatic nerve autograft. Fifty-two days after surgery, electrophysiology was performed and compound muscle action potentials were recorded in (**A**) the gastrocnemius muscle and (**B**) the tibialis anterior muscle. Four parameters were quantified from the CMAP traces: amplitude, peak AUC, take-off latency, and peak latency. Data are mean ± sem. N = 5–6 per group. One-way ANOVA with Šídák’s post hoc test was used. * *p* <  0.05, ** *p* <  0.01, *** *p* < 0.001, and **** *p* < 0.0001.

Gap43 mean intensity displayed a trend (*p* = 0.0778) toward an increase in the minocycline group compared to vehicle-treated animals (Figure 5A). Interestingly, animals that underwent the autograft procedure and were treated with minocycline displayed higher mean intensity for SOX10 in the common peroneal compared to vehicle-treated animals (Figure 5B). For the gastrocnemius muscle, the amplitude and the peak AUC were significantly higher in minocycline-treated animals compared with the vehicle group (1.9-fold increase in amplitude and 1.79-fold increase in peak AUC) (Figure 6A).

Minocycline-treated animals had lower latency values than vehicle-treated animals (1.34-fold decrease in take-off latency and 1.39-fold decrease in peak latency) (Figure 6A). Tibialis anterior muscle CMAP recordings showed an effect of the surgical procedure (sciatic nerve autograft) but no effect of the minocycline treatment compared with the vehicle-treated group for three parameters (amplitude, peak AUC, and peak latency) (Figure 6B), with take-off latency lower in the minocycline group compared with vehicle-treated animals (Figure 6B).

## 3. Discussion

Minocycline has been widely studied for its immunomodulatory and anti-inflammatory effects in vitro and in vivo and has shown beneficial effects in several animal models of CNS pathologies [8,9]. Its effects on the peripheral nervous system remained mostly unexplored. We set out to study minocycline’s effects in the context of peripheral nerve regeneration and its impact on Schwann cells, as they are a central player in the different processes occurring during nerve regeneration. One central feature of Schwann cells is their ability to switch toward a repair phenotype following nerve injury. We showed in vitro that minocycline upregulated several markers associated with the Schwann cell repair phenotype. For instance, N-cadherin is an important cell adhesion protein involved in Schwann cell–Schwann cell and Schwann cell–axon interactions and is necessary for axon guidance [31]. c-Jun is considered one of the main transcription factors responsible for the activation of the repair program in Schwann cells [32,33,34]. This master regulator is able to control the reprogramming of Schwann cells, supporting neuron survival, and promoting axonal regeneration [32,33]. In addition to the effects reported here on Schwann cell repair phenotype, minocycline was shown previously to protect Schwann cells from oxygen or glucose deprivation damage in vitro at 10 and 100 µM [11].

ROBO1 is a receptor expressed by Schwann cells that is another marker of the repair phenotype. One of its ligands, SLIT3, has been shown to be expressed in white adipose tissue macrophages in mice and increased in M2 macrophages. The authors further show that SLIT3 is able to increase thermogenesis through the activation of the sympathetic nervous system in white adipose tissue [35]. This put forth the importance of the crosstalk between macrophages and the peripheral nervous system in tissue homeostasis in the white adipose tissue and could also play a role in the context of nerve regeneration. Immune cell–Schwann cell interaction is an important aspect of the nerve regeneration process, although its full extent and the precise timing necessary for a good outcome still remain to be fully understood. The use of single-cell transcriptomic analysis in the context of nerve injury is unravelling novel cell subtypes and cell interactions, revealing potential new pharmacological targets to improve nerve regeneration [36].

In vivo, minocycline has been studied previously in peripheral nerves, mostly in the context of neuropathic pain and hyperalgesia, and was administered intrathecally, intramuscularly, or systemically [25,37,38]. In our two in vivo experiments, we showed that the local delivery of minocycline led to improved nerve regeneration and functional recovery. We elected to use local delivery in fibrin, as this approach is feasible in the clinic during nerve repair surgery and is an effective way to avoid potential systemic side effects. The approach of repurposing an approved drug and delivering it using a material already used in nerve surgery clearly presents great potential for clinical translation. One previous study that evaluated the systemic delivery of minocycline for seven days in a sciatic nerve gap repair model found that this treatment was detrimental to nerve regeneration due to its effects on macrophage recruitment and activation [10]. These deleterious effects could be explained by the route of administration and the dose regimen since minocycline was administered systemically by daily intra-peritoneal injection, which could lead to profound changes in the activation of circulating immune cells, such as monocytes or T-cells. The same group observed the detrimental effects of minocycline in a second study using a sciatic nerve gap repair but found that systemic minocycline was able to improve the axon count in the distal nerve when the graft used for the repair consisted of a mix of muscle grafts and Schwann cells [11].

We showed in the two in vivo experiments (sciatic nerve transection direct repair and autograft repair) that nerve regeneration was improved. In the common peroneal nerve, we found an increase in GAP43. This protein has been shown to increase the growth potential of injured neurons, and its expression was associated with the regeneration capacity of reimplanted spinal nerves in a brachial plexus root avulsion model. Additionally, its increase has been correlated with functional recovery [39,40,41]. In our experimental setting, the GAP43 increase could indicate an increase in sprouting, and this should be investigated in future work.

In the sciatic nerve autograft experiment, minocycline local delivery led to an increase in SOX10 in the common peroneal nerve. This transcription factor is necessary for Schwann cell homeostasis and for myelination maintenance in the intact peripheral nervous system [42]. In the context of nerve regeneration, SOX10 activates the transcription of genes involved in myelination and regulates Schwann cell proliferation [29,30]. For both nerve regeneration models, minocycline local delivery led to improved functional outcomes and specifically for four parameters extracted from the gastrocnemius muscle CMAP traces. Of course, it is difficult to directly compare the two animal models used (different types of injury, different times of treatment, different duration) but it is interesting to note that SOX10 was increased in the common peroneal nerve of rats that underwent the sciatic nerve autograft (fifty-two days after surgery and treatment) and not in the rats that underwent the sciatic nerve transection (thirty-five days after surgery, twenty-eight days after treatment). The SOX10 increase measured in the common peroneal nerve of minocycline-treated autografted animals could be ascribed to a higher production of the protein and/or an increase in Schwann cell proliferation.

This increase was associated with an improvement in the take-off latency in the autograft experiment for rats treated with minocycline. This was associated with improvements in motor functions as measured with CMAP parameters. CMAP is considered the most suitable parameter to assess functional recovery, as it reflects motor axon regeneration and muscle reinnervation [43]. As highlighted by the SOX10 increase and CMAP improvements, processes involved in remyelination could be involved in the beneficial effects of minocycline, even though we observed no significant change in MBP. It is worth noting that we assessed these markers at one location in the sciatic nerve and in the common peroneal nerve. They could display variation at other sites of the nerve. Indeed, nerve regeneration and remyelination are two dynamic processes that depend on the type and location of injury. Future studies should explore these factors in multiple locations and at a variety of time points to better identify minocycline’s effects on remyelination.

Minocycline treatment led to an increase of the CMAP amplitude by 322% compared with the vehicle-treated rats in the transection model and to an increase of 96% in the autograft model. Other pharmacological treatments have been tested in order to improve nerve regeneration but did not reach that level of benefit in terms of functional improvement. For instance, in a sciatic nerve crush model, several PPARγ ligands were tested and, after twenty-eight days, the best observed effect on gastrocnemius CMAP amplitude was 75% compared to vehicle-treated rats [44].

We showed that minocycline is able to promote markers associated with the repair phenotype in Schwann cells in vitro. However, this compound is also able to act on many other cell types, including immune cells. Therefore, the beneficial effects we observed in nerve regeneration and functional recovery cannot simply be ascribed to Schwann cells alone. Interestingly, some of the repair phenotype markers that were increased by minocycline in vitro on SCL 4.1/F7 Schwann cells are also known to be important in the crosstalk between Schwann cells and immune cells. Indeed, SOX2 has been found to be able to control monocyte/macrophage recruitment in the nerve in an experiment using mice overexpressing SOX2 [45]. The authors also found a trend toward an increase of T-cells in the same animals. It would be interesting to investigate the effects of minocycline on T-cells and macrophages in both in vivo models (sciatic nerve transection and sciatic nerve autograft). However, in this study, we focused on potential changes in motor function and, therefore, we used later time points (thirty-five and fifty-two days) after surgery, whereas to assess minocycline effects on T-cells and macrophages, it would be more appropriate to use earlier time points.

The exact mechanism(s) mediating minocycline’s effects remain to be fully identified but it is clear from the present study that it has some potential to improve functional outcomes when administered locally following nerve transection and repair.

In this study, we showed that minocycline in vitro was able to change the expression of markers associated with the phenotype of Schwann cells toward their repair program. We also showed, using two in vivo models, that local delivery of minocycline using fibrin gel was able to significantly improve nerve regeneration and functional outcome. These results represent a very interesting avenue to explore as there is currently no pharmacological approach available to improve nerve regeneration, and the delivery approach used would be easily translated to the clinic. In the future, it would be interesting to explore the mechanisms and cell types involved in the beneficial effects observed and to fine-tune the dose delivered locally.

## 4. Materials and Methods

### 4.1. Cell Culture

RAW 264.7 cells (mouse macrophage-like cell line) and SCL 4.1/F7 cells (rat Schwann cell line) were cultured using high-glucose DMEM medium (Sigma, Gillingham, UK. ref: D6429) supplemented with 10% heat-inactivated FBS (Gibco, Paisley, UK. ref: 10500056) and antibiotics (100 U/mL penicillin and 100 μg/mL streptomycin (Sigma, Gillingham, UK. ref: P0781)). All cell cultures and subsequent experiments were maintained under standard conditions of 37 °C in a humidified 5% CO_2_ incubator).

Cells were seeded in 24-well plates or 384-well plates, and after 12 h, media was changed and fresh media containing vehicle (water) or minocycline (Sigma, Gillingham, UK. ref M9511) at 1 μM, 20 μM, or 40 μM was added for 24 h. Cells were subsequently used for RT-qPCR or immunofluorescence and RAW 264.7 media was used for ELISA.

### 4.2. Sciatic Nerve Explants

Sciatic nerves were harvested from Sprague Dawley rats and cultured using high-glucose DMEM medium (Sigma, Gillingham, UK. ref: D6429) supplemented with 10% heat-inactivated FBS (Gibco, Paisley, UK. ref: 10500056) and antibiotics (100 U/mL penicillin and 100 μg/mL streptomycin (Sigma, Gillingham, UK. ref: P0781)). They were maintained under standard conditions of 37 °C in a humidified 5% CO_2_ incubator) during the duration of the experiments.

Sciatic nerves were cut into 0.5 cm segments (for immunofluorescence experiments) or 1 cm segments (for PCR experiments) upon harvest. After 12 h, the media was changed and fresh media containing vehicle (water) or minocycline (Sigma, Gillingham, UK. ref M9511) at 1 μM or 40 μM was added for 24 h. Tissues were subsequently used for RT-qPCR or immunofluorescence.

### 4.3. RT-qPCR

Total RNA was extracted using the RNeasy Plus Mini Kit (Qiagen, Manchester, UK. ref: 74136) according to the manufacturer’s instructions. cDNA was synthesised using the GoScript Reverse Transcriptase kit (Promega, Southampton, UK. ref: A2791). RT-qPCR was performed with a QuantStudio 3 instrument (Applied Biosystems, Waltham, MA, USA) and analysed with QuantStudio Design and Analysis Software v1.5.1(Applied Biosystems). PCR reactions were run in duplicate using the Power SYBR Green PCR Master Mix (Thermo, Paisley, UK. ref: 4368708). The amplification products were analysed by performing a melting curve at the end of the PCR. Data were normalised to the mRNA expression of three reference genes: hypoxanthine guanine phosphoribosyl transferase (*Hprt1*), ribosomal protein L19 (*Rpl19*), and TATA box binding protein (*Tbp*) for RAW 264.7 cells (mouse) as well as beta-2 microglobulin (*B2m*), hypoxanthine phosphoribosyltransferase 1 (*Hprt1*), and ribosomal protein S18 (*Rps18*) for SCL 4.1/F7 cells (rat). The expression of the reference genes was not affected by the experimental conditions. Primer sequences for RT-qPCR (mouse and rat) are listed in Table 1.

### 4.4. CCL2 Quantification by ELISA

CCL2 levels were quantified in RAW 264.7 cell media after 24 h incubation with vehicle (water) or minocycline (Sigma, Gillingham, UK. ref M9511) at 1 μM, 20 μM, or 40 μM. A sandwich-type ELISA kit (Invitrogen, Paisley, UK. ref: 88-7391-88) was used according to the manufacturer’s instructions.

### 4.5. Nerve Regeneration Models

All surgical procedures were performed in accordance with the UK Animals (Scientific Procedures) Act (1986) and the European Communities Council Directives (86/609/EEC), and approved by the UCL Animal Welfare and Ethics Review Board (AWERB). Adult male Wistar rats (250–300 g) (Charles River, Wilmington, MA, USA) were housed in a controlled environment (12 h day–light cycle, controlled temperature, and humidity) with free access to food and water. Upon arrival, they were randomised into groups and acclimated for a week. Anaesthesia was performed using isoflurane (5% for induction and 3% for maintenance) and the animals were monitored throughout the procedure to ensure depth of anaesthesia. Under a microscope (Zeiss CL 1500 ECO, Carl Zeiss GmbH, Cambridge, UK), the sciatic nerve was released from the surrounding tissue.

For the first in vivo experiment, a sciatic nerve transection was performed at day 0 and the nerve stumps were sutured back using two 10/0 sutures. Seven days after the transection, a fibrin gel (Tisseel 2 mL, Baxter, Thetford, UK) containing water (vehicle, *n* = 5) or minocycline (*n* = 7) was applied directly to the repaired transection site. Technically, 10 μL of thrombin was applied to the surface of the sciatic nerve and 20 μL of a 1:1 mix of either water/fibrinogen or minocycline/fibrinogen was then applied and left to set for 5 min. An amount of 100 μg of minocycline was administered based on doses typically used in the central nervous system [25,26,27]. Thirty-five days after the sciatic nerve transection, electrophysiology recordings were performed, and ipsilateral sciatic nerves and common peroneal nerves were harvested.

For the second in vivo experiment, a sciatic nerve autograft (1 cm) was performed on day 0. A segment of 1 cm was excised and reversed before being sutured back (two 10/0 sutures per site). Fibrin gel (Tisseel 2 mL, Baxter) containing water (vehicle, *n* = 5) or minocycline (*n* = 6) was applied directly to the two sutured sites. Technically, 5 μL of thrombin was applied to each suture site and 10 μL of a 1:1 mix of either water/fibrinogen or minocycline/fibrinogen was then applied and left to set for 5 min. An amount of 100 μg of minocycline was administered per sciatic nerve or 50 μg per suture site. Fifty-two days after the autograft, electrophysiology recordings were performed, and ipsilateral sciatic nerves and common peroneal nerves were harvested.

### 4.6. Electrophysiology Recordings

Electrophysiological tests were performed to assess muscle reinnervation 35 days post-transection for the first experiment and 52 days post-autograft for the second experiment. Animals were maintained under terminal anaesthesia on a heated operating table. Motor nerve stimulation and CMAP recordings were performed using a Dantec^®^ Keypoint*^®^* Focus EMG/NCS/EP System (Natus Keypoint, Bicester, UK., ref 9033A07) and Synergy Electrodiagnostic Software v22.3.0.21 (Natus neuro 2019, Bicester, UK) in both ipsi- and contra-lateral sides. The stimulating electrode (using a bipolar stimulation constant voltage configuration) was positioned 0.2 cm proximal from the sciatic nerve transection (transection model) or 0.2 cm proximal from the first (most proximal) sciatic nerve transection (autograft model), and a recording electrode was placed into the gastrocnemius muscle or the tibialis anterior muscle. The grounding electrode was positioned in the rat’s tail and a reference electrode was placed above the hip bone. The stimulation threshold was determined by increasing the stimulus amplitude in 0.1 V steps until both a supra-maximal muscle action potential was recorded and a significant twitch of the animal’s hind paw was observed. The compound muscle action potential (CMAP) of the tibialis anterior and gastrocnemius muscles was recorded. The amplitude, peak AUC, take-off latency, and peak latency were measured.

### 4.7. Immunofluorescence

SCL 4.1/F7 cells were cultured in a 384-well plate and used after 24 h of incubation in media containing minocycline 1 μM or 40 μM or vehicle (water). Cells were fixed for 16 h in a buffered solution of 4% formaldehyde (Sigma-Aldrich, ref: 1004969011) and washed with PBS. Primary antibodies: ROBO1 (1/200, Abcam, Cambridge, UK. ref: Ab7279), NCAD (1/200, Abcam ab18203), and c-Jun (1/300 Cell Signaling, London, UK. ref 9165) were incubated for 24 h at 4 °C. Cells were washed three times in PBS and incubated for 24 h at 4 °C with the relevant secondary antibodies and Hoechst 33342 (Thermo, Ref H3570): Goat anti-Rabbit 549 (1/300, DyLight DI-1549) and Goat anti-Mouse 488 (Alexa Fluor + A32723). Then, 384-well plates were scanned on an Opera Phenix (Perkin Elmer, Beaconsfield, UK) and analysed using Columbus image analysis software v 2.9.1 (Perkin Elmer).

Sciatic nerves and common peroneal nerves were harvested and fixed in 4% PFA for 24 h, transferred in sucrose solutions (15% then 30%) for cryoprotection, and embedded in OCT and frozen. Cryosections (10 μm) were cut with a cryostat (Thermo Scientific HM525). Serial cutting was performed, resulting in five nerve slices per slide, and a total of five slides were used. For the first experiment (transection with direct repair), sciatic nerves were cryosectioned (10 μm) 0.7 cm distal from the transection site and the common peroneal nerves at their muscle insertion. For the second experiment (autograft), the common peroneal nerves were cryosectioned (10 μm) at their muscle insertion. Primary antibodies: GAP-43 (1/300, Millipore, Gillingham, UK. ref AB5220), Neurofilament 488-conjugated (1/500, Biolegend 835604), c-Jun (1/200, Cell Signaling 9165S), SOX10 (1/200, R&D Systems MAB2864), and MBP (1/2000, Invitrogen PA1-10008) were incubated for 24 h at 4 °C. Tissues were washed three times in PBS and incubated for 24 h at 4 °C with the relevant secondary antibodies (1/300) and Hoechst 33342 (Thermo, Ref H3570): Goat anti-Rabbit 647 (Alexa Fluor + A32733), Goat anti-Rabbit 555 (Alexa Fluor + A32732), Goat anti-Mouse 488 (Alexa Fluor + A32723), and Goat anti-Chicken 647 (Alexa Fluor + A32933). Omission of primary or secondary antibodies was used as a control. Slides were scanned on a NanoZoomer S60 Digital slide scanner (Hamamatsu, Japan). For each marker, the average of the five nerve slices per slide was quantified. Axon count (neurofilament) was measured using Volocity software v6.5.1 (PerkinElmer, Beaconsfield, UK). The remaining markers were quantified using Fiji ImageJ [46]. The nerve section was selected and the intensity of all pixels was measured and averaged to obtain the mean intensity for each marker.

### 4.8. Statistical Analysis

All data are presented as mean  ±  SEM. Statistical analysis was performed using GraphPad Prism version 9.0. The Kolmogorov–Smirnov test was used to assess the normality of the distribution. A two-tailed unpaired *t*-test or a Mann–Whitney test was used for comparing the two groups. A one-way ANOVA with Dunnett’s post hoc or a Kruskal–Wallis with Dunn’s post hoc test was used except for the electrophysiology results, where a one-way ANOVA with Šídák’s post hoc test was used. * *p* <  0.05, ** *p* <  0.01, *** *p* < 0.001, and **** *p* < 0.0001, and a trend was suggested when *p* < 0.1 and the exact *p*-value was displayed on the graph.

## Figures and Tables

**Table 1:** Primer sequence.

Species	Name	Symbol	Forward Primer (5′-3′)	Reverse Primer (5′-3′)
Mouse	chemokine (C-C motif) ligand 2	*Ccl2*	GTCCCAAAGAAGCTGTAGTTTTTG	ATGTATGTCTGGACCCATTCC
Mouse	chemokine (C-C motif) ligand 3	*Ccl3*	AGATTCCACGCCAATTCATC	CTCAAGCCCCTGCTCTACAC
Mouse	chemokine (C-X-C motif) ligand 2	*Cxcl2*	TCAACGGAAGAACCAAAGAG	AATAAGTGAACTCTCAGACAGC
Mouse	hypoxanthine guanine phosphoribosyl transferase	*Hprt1*	GACTGAAGAGCTACTGTAATG	AGATCATCTCCACCAATAAC
Mouse	prostaglandin-endoperoxide synthase 2	*Ptgs2*	TGACCCCCAAGGCTCAAATAT	TGAACCCAGGTCCTCGCTTA
Mouse	ribosomal protein L19	*Rpl19*	TGACCTGGATGAGAAGGATGAG	CTGTGATACATATGGCGGTCAATC
Mouse	TATA box binding protein	*Tbp*	CCCTTCACCAATGACTCCTATG	ACAGCCAAGATTCACGGTAG
Mouse	tumor necrosis factor	*Tnf*	CTACTGAACTTCGGGGTGATC	TGAGTGTGAGGGTCTGGGC
Rat	beta-2 microglobulin	*B2m*	CGTGATCTTTCTGGTGCTTG	GGTGGAACTGAGACACGTAG
Rat	C-C motif chemokine ligand 2	*Ccl2*	GCAAGATGATCCCAATGAGTC	GCTTGGTGACAAATACTACAGC
Rat	glial cell derived neurotrophic factor	*Gdnf*	GCTGACCAGTGACTCCAATATG	TGCCGCCGCTTGTTTATC
Rat	hypoxanthine phosphoribosyltransferase 1	*Hprt1*	ACCTCTCGAAGTGTTGGATAC	GATTCAAATCCCTGAAGTGCTC
Rat	LIF, interleukin 6 family cytokine	*Lif*	CAAGAGTCAACTGGCTCAAC	GCATGGAAAGGTGGGAAATC
Rat	neural cell adhesion molecule 1	*Ncam1*	GTGAGGTCTTTGCCTACC	CCAGATAGCTCGCAGATG
Rat	roundabout guidance receptor 1	*Robo1*	CTGGACGGAAGACAAGTCAC	GGTGGAAGGTCTCGCTTTG
Rat	ribosomal protein S18	*Rps18*	CTTCGCTATCACTGCCATTAAG	GTGAGGTCAATGTCTGCTTTC
Rat	SRY-box transcription factor 2	*Sox2*	GCAGTACAACTCCATGACCAG	GCGAGTAGGACATGCTGTAG

## Data Availability

The authors confirm that the data supporting the findings of this study are available within the article and its Supplementary Materials.

## Supplementary Materials

The supporting information can be downloaded at: https://www.mdpi.com/article/10.3390/ijms241512085/s1.

## References

[1] Ruijs A.C., Jaquet J.B., Kalmijn S., Giele H., Hovius S.E. (2005). Median and ulnar nerve injuries: A meta-analysis of predictors of motor and sensory recovery after modern microsurgical nerve repair. Plast. Reconstr. Surg..

[2] Pfister B.J., Gordon T., Loverde J.R., Kochar A.S., Mackinnon S.E., Cullen D.K. (2011). Biomedical engineering strategies for peripheral nerve repair: Surgical applications, state of the art, and future challenges. Crit. Rev. Biomed. Eng..

[3] Grinsell D., Keating C.P. (2014). Peripheral nerve reconstruction after injury: A review of clinical and experimental therapies. Biomed. Res. Int..

[4] Sameem M., Wood T.J., Bain J.R. (2011). A systematic review on the use of fibrin glue for peripheral nerve repair. Plast. Reconstr. Surg..

[5] Koopman J.E., Duraku L.S., de Jong T., de Vries R.B.M., Michiel Zuidam J., Hundepool C.A. (2022). A systematic review and meta-analysis on the use of fibrin glue in peripheral nerve repair: Can we just glue it?. J. Plast. Reconstr. Aesthetic Surg..

[6] Tikka T.M., Koistinaho J.E. (2001). Minocycline provides neuroprotection against N-methyl-D-aspartate neurotoxicity by inhibiting microglia. J. Immunol..

[7] Vandooren J., Knoops S., Aldinucci Buzzo J.L., Boon L., Martens E., Opdenakker G., Kolaczkowska E. (2017). Differential inhibition of activity, activation and gene expression of MMP-9 in THP-1 cells by azithromycin and minocycline versus bortezomib: A comparative study. PLoS ONE.

[8] Garrido-Mesa N., Zarzuelo A., Galvez J. (2013). What is behind the non-antibiotic properties of minocycline?. Pharmacol. Res..

[9] Yong V.W., Wells J., Giuliani F., Casha S., Power C., Metz L.M. (2004). The promise of minocycline in neurology. Lancet Neurol..

[10] Keilhoff G., Langnaese K., Wolf G., Fansa H. (2007). Inhibiting effect of minocycline on the regeneration of peripheral nerves. Dev. Neurobiol..

[11] Keilhoff G., Schild L., Fansa H. (2008). Minocycline protects Schwann cells from ischemia-like injury and promotes axonal outgrowth in bioartificial nerve grafts lacking Wallerian degeneration. Exp. Neurol..

[12] Jessen K.R., Mirsky R. (2016). The repair Schwann cell and its function in regenerating nerves. J. Physiol..

[13] Ydens E., Amann L., Asselbergh B., Scott C.L., Martens L., Sichien D., Mossad O., Blank T., De Prijck S., Low D. (2020). Profiling peripheral nerve macrophages reveals two macrophage subsets with distinct localization, transcriptome and response to injury. Nat. Neurosci..

[14] Cattin A.L., Burden J.J., Van Emmenis L., Mackenzie F.E., Hoving J.J., Garcia Calavia N., Guo Y., McLaughlin M., Rosenberg L.H., Quereda V. (2015). Macrophage-Induced Blood Vessels Guide Schwann Cell-Mediated Regeneration of Peripheral Nerves. Cell.

[15] Spicer P.P., Mikos A.G. (2010). Fibrin glue as a drug delivery system. J. Control. Release.

[16] Wood M.D., Gordon T., Kim H., Szynkaruk M., Phua P., Lafontaine C., Kemp S.W., Shoichet M.S., Borschel G.H. (2013). Fibrin gels containing GDNF microspheres increase axonal regeneration after delayed peripheral nerve repair. Regen. Med..

[17] Liu F.Y., Wu Y.H., Zhou S.J., Deng Y.L., Zhang Z.Y., Zhang E.L., Huang Z.Y. (2014). Minocycline and cisplatin exert synergistic growth suppression on hepatocellular carcinoma by inducing S phase arrest and apoptosis. Oncol. Rep..

[18] Filipovic R., Zecevic N. (2008). Neuroprotective role of minocycline in co-cultures of human fetal neurons and microglia. Exp. Neurol..

[19] Choi Y., Kim H.S., Shin K.Y., Kim E.M., Kim M., Kim H.S., Park C.H., Jeong Y.H., Yoo J., Lee J.P. (2007). Minocycline attenuates neuronal cell death and improves cognitive impairment in Alzheimer’s disease models. Neuropsychopharmacology.

[20] Kobayashi K., Imagama S., Ohgomori T., Hirano K., Uchimura K., Sakamoto K., Hirakawa A., Takeuchi H., Suzumura A., Ishiguro N. (2013). Minocycline selectively inhibits M1 polarization of microglia. Cell Death Dis..

[21] Amin A.R., Attur M.G., Thakker G.D., Patel P.D., Vyas P.R., Patel R.N., Patel I.R., Abramson S.B. (1996). A novel mechanism of action of tetracyclines: Effects on nitric oxide synthases. Proc. Natl. Acad. Sci. USA.

[22] Kohno H., Chen Y., Kevany B.M., Pearlman E., Miyagi M., Maeda T., Palczewski K., Maeda A. (2013). Photoreceptor proteins initiate microglial activation via Toll-like receptor 4 in retinal degeneration mediated by all-trans-retinal. J. Biol. Chem..

[23] Jessen K.R., Mirsky R. (2019). The Success and Failure of the Schwann Cell Response to Nerve Injury. Front. Cell. Neurosci..

[24] Chen Q., Liu Q., Zhang Y., Li S., Yi S. (2021). Leukemia inhibitory factor regulates Schwann cell proliferation and migration and affects peripheral nerve regeneration. Cell Death Dis..

[25] Mei X.P., Xu H., Xie C., Ren J., Zhou Y., Zhang H., Xu L.X. (2011). Post-injury administration of minocycline: An effective treatment for nerve-injury induced neuropathic pain. Neurosci. Res..

[26] Hua X.Y., Svensson C.I., Matsui T., Fitzsimmons B., Yaksh T.L., Webb M. (2005). Intrathecal minocycline attenuates peripheral inflammation-induced hyperalgesia by inhibiting p38 MAPK in spinal microglia. Eur. J. Neurosci..

[27] Ismail C.A.N., Suppian R., Aziz C.B.A., Long I. (2019). Minocycline attenuates the development of diabetic neuropathy by modulating DREAM and BDNF protein expression in rat spinal cord. J. Diabetes Metab. Disord..

[28] Carriel V., Garzon I., Campos A., Cornelissen M., Alaminos M. (2017). Differential expression of GAP-43 and neurofilament during peripheral nerve regeneration through bio-artificial conduits. J. Tissue Eng. Regen. Med..

[29] Fujiwara S., Hoshikawa S., Ueno T., Hirata M., Saito T., Ikeda T., Kawaguchi H., Nakamura K., Tanaka S., Ogata T. (2014). SOX10 transactivates S100B to suppress Schwann cell proliferation and to promote myelination. PLoS ONE.

[30] Hung H.A., Sun G., Keles S., Svaren J. (2015). Dynamic regulation of Schwann cell enhancers after peripheral nerve injury. J. Biol. Chem..

[31] Wanner I.B., Wood P.M. (2002). N-cadherin mediates axon-aligned process growth and cell-cell interaction in rat Schwann cells. J. Neurosci..

[32] Arthur-Farraj P.J., Latouche M., Wilton D.K., Quintes S., Chabrol E., Banerjee A., Woodhoo A., Jenkins B., Rahman M., Turmaine M. (2012). c-Jun reprograms Schwann cells of injured nerves to generate a repair cell essential for regeneration. Neuron.

[33] Fontana X., Hristova M., Da Costa C., Patodia S., Thei L., Makwana M., Spencer-Dene B., Latouche M., Mirsky R., Jessen K.R. (2012). c-Jun in Schwann cells promotes axonal regeneration and motoneuron survival via paracrine signaling. J. Cell Biol..

[34] Wagstaff L.J., Gomez-Sanchez J.A., Fazal S.V., Otto G.W., Kilpatrick A.M., Michael K., Wong L.Y.N., Ma K.H., Turmaine M., Svaren J. (2021). Failures of nerve regeneration caused by aging or chronic denervation are rescued by restoring Schwann cell c-Jun. Elife.

[35] Wang Y.N., Tang Y., He Z., Ma H., Wang L., Liu Y., Yang Q., Pan D., Zhu C., Qian S. (2021). Slit3 secreted from M2-like macrophages increases sympathetic activity and thermogenesis in adipose tissue. Nat. Metab..

[36] Lovatt D., Tamburino A., Krasowska-Zoladek A., Sanoja R., Li L., Peterson V., Wang X., Uslaner J. (2022). scRNA-seq generates a molecular map of emerging cell subtypes after sciatic nerve injury in rats. Commun. Biol..

[37] Cheng K.I., Wang H.C., Wu Y.C., Tseng K.Y., Chuang Y.T., Chou C.W., Chen P.L., Chang L.L., Lai C.S. (2016). Sciatic Nerve Intrafascicular Lidocaine Injection-induced Peripheral Neuropathic Pain: Alleviation by Systemic Minocycline Administration. Clin. J. Pain.

[38] Dunn J.S., Nagi S.S., Mahns D.A. (2020). Minocycline reduces experimental muscle hyperalgesia induced by repeated nerve growth factor injections in humans: A placebo-controlled double-blind drug-crossover study. Eur. J. Pain.

[39] Zhang Y., Xu L., Li X., Chen Z., Chen J., Zhang T., Gu X., Yang J. (2022). Deciphering the dynamic niches and regeneration-associated transcriptional program of motoneurons following peripheral nerve injury. iScience.

[40] Chung D., Shum A., Caraveo G. (2020). GAP-43 and BASP1 in Axon Regeneration: Implications for the Treatment of Neurodegenerative Diseases. Front. Cell Dev. Biol..

[41] Yuan Q., Hu B., Su H., So K.F., Lin Z., Wu W. (2009). GAP-43 expression correlates with spinal motoneuron regeneration following root avulsion. J. Brachial Plex. Peripher. Nerve Inj..

[42] Bremer M., Frob F., Kichko T., Reeh P., Tamm E.R., Suter U., Wegner M. (2011). Sox10 is required for Schwann-cell homeostasis and myelin maintenance in the adult peripheral nerve. Glia.

[43] Navarro X. (2016). Functional evaluation of peripheral nerve regeneration and target reinnervation in animal models: A critical overview. Eur. J. Neurosci..

[44] Rayner M.L.D., Kellaway S.C., Kingston I., Guillemot-Legris O., Gregory H., Healy J., Phillips J.B. (2022). Exploring the Nerve Regenerative Capacity of Compounds with Differing Affinity for PPARgamma In Vitro and In Vivo. Cells.

[45] Roberts S.L., Dun X.P., Doddrell R.D.S., Mindos T., Drake L.K., Onaitis M.W., Florio F., Quattrini A., Lloyd A.C., D’Antonio M. (2017). Sox2 expression in Schwann cells inhibits myelination in vivo and induces influx of macrophages to the nerve. Development.

[46] Schindelin J., Arganda-Carreras I., Frise E., Kaynig V., Longair M., Pietzsch T., Preibisch S., Rueden C., Saalfeld S., Schmid B. (2012). Fiji: An open-source platform for biological-image analysis. Nat. Methods.

