# Supplementary material for: Local Administration of Minocycline Improves Nerve Regeneration in Two Rat Nerve Injury Models
Source: Int J Mol Sci. 2023 Jul 28;24(15):12085. doi: 10.3390/ijms241512085 (PMC10418394; doi:10.3390/ijms241512085)
Supplement: Supplementary file 1 [file ijms-24-12085-s001.zip › ijms-2483001-supplementary.pdf]

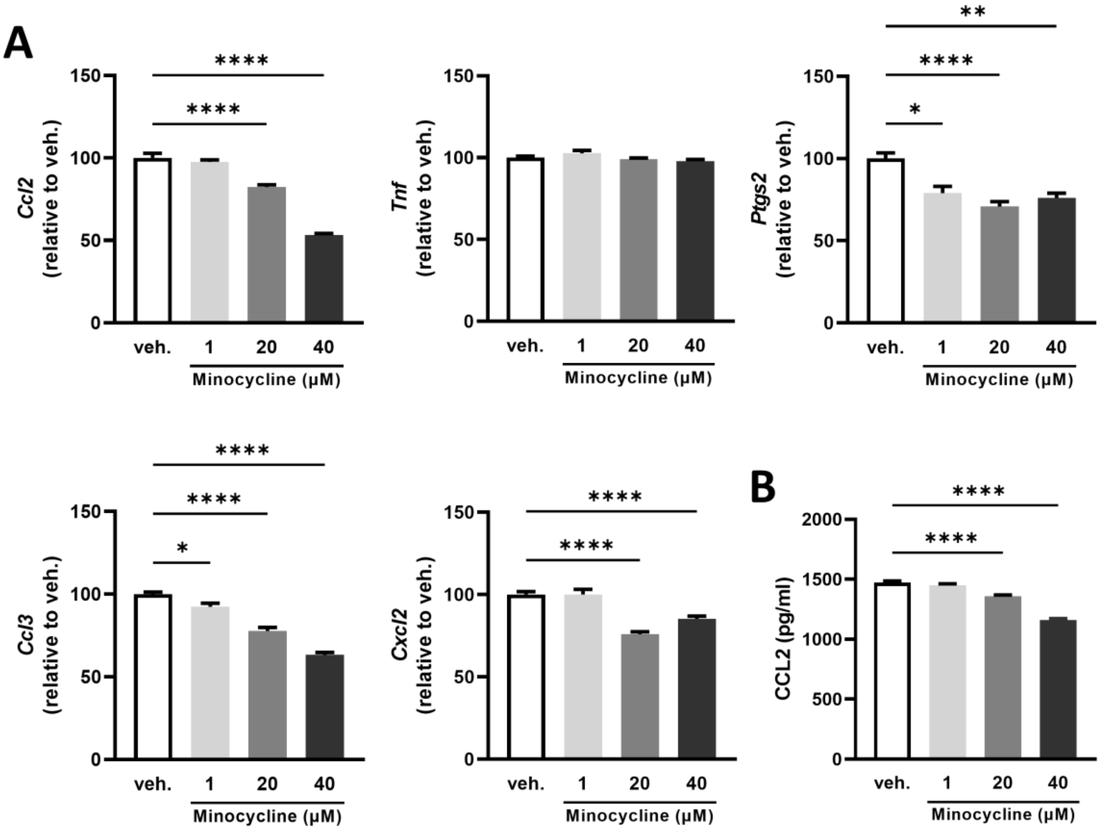

Figure S1 Minocycline decreases RAW 264.7 macrophage activation. (**A**) mRNA expression of *Ccl2*, *Tnf*, *Ptgs2*, *Ccl3*, and *Cxcl2* in RAW 264.7 cells treated with minocycline (1, 20, 40 µM) or vehicle (veh.) for 24h assessed by RT-qPCR. The expression of the control group (veh.) was set at 100. (**B**) CCL2 levels were quantified in RAW 264.7 media by ELISA after 24h incubation with minocycline (1, 20, 40 µM) or vehicle (veh.). Data are mean ± sem. N=3 in quadruplicate. One-way ANOVA with Dunnett's post-hoc test or Kruskal-Wallis with Dunn's post-hoc test depending on the normality of the distribution. *P < 0.05, **P < 0.01, and **** P<0.0001.

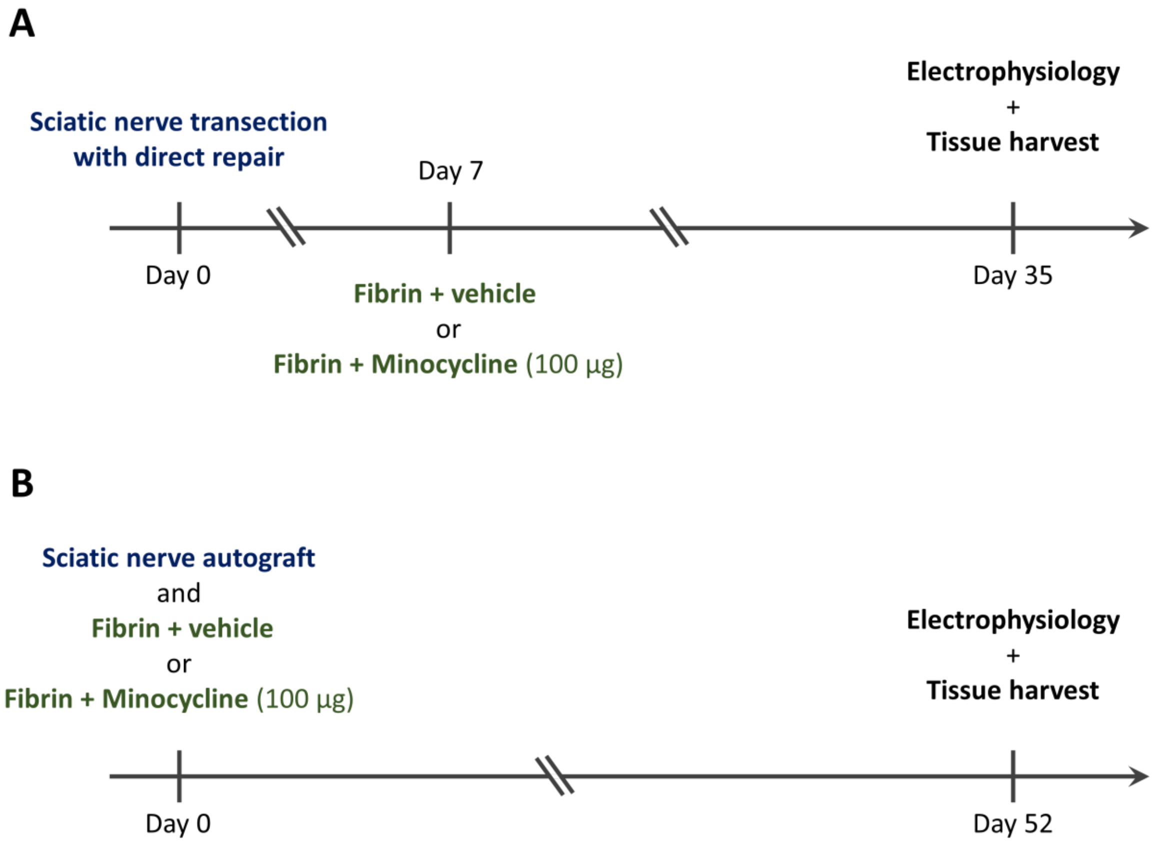

Figure S2 Timeline of the two nerve regeneration experiments. (**A**) Time lime of the sciatic nerve transection experiment. Rats underwent a sciatic nerve transection and the stumps were sutured directly on day 0. After seven days, fibrin containing water (vehicle) or fibrin containing 100µg of minocycline was applied at the transection repair site. Thirty-five days after the transection (or twenty-eight days after treatment), electrophysiology was performed and tissues harvested. (**B**) Timeline of the sciatic nerve autograft. Wistar rats underwent a sciatic nerve autograft (1 cm gap) and fibrin containing water (vehicle) or fibrin containing 100µg of minocycline was applied to the two suture sites (50 µg per site, total of 100 µg per nerve). Fifty-two days after the surgery, electrophysiology was performed and tissues harvested.
